# Significance of CD10 for Mucosal Immunomodulation by β-Casomorphin-7 in Exacerbation of Ulcerative Colitis

**DOI:** 10.3390/cimb46070386

**Published:** 2024-06-26

**Authors:** Yoshihiro Miyagawa, Rina Fujiwara-Tani, Ayaka Ikemoto, Rika Sasaki, Ruiko Ogata, Yukiko Nishiguchi, Kei Goto, Isao Kawahara, Takamitsu Sasaki, Hiroki Kuniyasu

**Affiliations:** Department of Molecular Pathology, Nara Medical University, 840 Shijo-cho, Kashihara 634-8521, Nara, Japan; y.miya1103@gmail.com (Y.M.); a.ikemoto.0916@gmail.com (A.I.); rika0st1113v726296v@icloud.com (R.S.); pkuma.og824@gmail.com (R.O.); yukko10219102@yahoo.co.jp (Y.N.); ilgfgtk@gmail.com (K.G.); isao_kawahara@a011.broada.jp (I.K.); takamitu@fc4.so-net.ne.jp (T.S.)

**Keywords:** inflammatory bowel diseases, ulcerative colitis, β-casomorphin-7, CD10, CD8+ T cell, antimicrobial peptide

## Abstract

β-Casomorphin-7 (BCM), a breakdown product of milk β-casein, exhibits opioid activity. Opioids are known to affect the immune system, but the effects of BCM on ulcerative colitis (UC) are not clear. We examined the effects of BCM on mucosal immunity using a mouse dextran sulfate sodium-induced colitis model and an in vitro CD8+ T cell activation model. Human UC patients were examined to reveal the relationship between CD10 and mucosal immunity. Combined treatment of the colitis model with thiorphan (TOP) inhibited BCM degradation by suppressing CD10 in the intestinal mucosa, activating mouse mucosal CD8, and suppressing CD4 and Treg. In the CD8+ T cell in vitro activation assay using mouse splenocytes, BCM inhibited the oxidative phosphorylation (OXPHOS) of CD8+ T cells and induced the glycolytic pathway, promoting their activation. Conversely, in a culture system, BCM suppressed OXPHOS and decreased defensin α production in IEC6 mouse intestinal epithelial cells. In the mouse model, BCM reduced defensin α and butyrate levels in the colonic mucosa. During the active phase of human ulcerative colitis, the downward regulation of ileal CD10 expression by CpG methylation of the gene promoter was observed, resulting in increased CD8 activation and decreased defensin α and butyrate levels. BCM is a potential aggravating factor for UC and should be considered in the design of dietary therapy. In addition, decreased CD10 expression may serve as an indicator of UC activity and recurrence, but further clinical studies are needed.

## 1. Introduction

Ulcerative colitis (UC) constitutes a heterogeneous condition characterized by chronic intestinal inflammation of unknown etiology [1]. UC is influenced by genetic and environmental factors, dysbiosis of the microbiota, decreased innate and adaptive immunity, and dysfunction of the intestinal epithelial barrier [1]. In 2023, the prevalence of ulcerative colitis was estimated to reach 5 million people worldwide [2]. The active phase of UC has a significant impact on the patient’s quality of life, and its exacerbation requires adequate management [3]. UC is an autoimmune disease, and immune-targeting drugs have been developed. Major advances in the treatment of UC have been made with aminosalicylates, immunomodulators, biologics, and orally targeted small-molecule inhibitors [4]. Cytokine-targeted therapy has been shown to be effective in treating UC patients, but responses are variable and may be due to differences in cytokine profiles among UC patients [5]. To overcome these differences in cytokine profiles, new drugs with various mechanisms of action have been developed. Several new biologics—mirizikizumab (IL-23 blocker), tofacitinib and upadacitinib (JAK inhibitors, ozanimod and etrasimod (sphingosine-1-phosphate receptor modulators))—have been approved for the treatment of moderate to severe ulcerative colitis, and these new agents add novel mechanisms of action to the expanding therapeutic tools of advanced therapies in the treatment of ulcerative colitis [6,7].

Understanding the factors that contribute to disease exacerbation is important for the management of UC. Nutritional management in UC has received a lot of attention [8,9,10]. Regarding milk intake, children with a history of cow’s milk protein allergy have a higher prevalence of UC [11]. However, dietary cow’s milk protein removal has no utility in the management of UC in non-sensitized children [12]. There is no difference in UC recurrence between dairy-free and control diets [13,14]. Based on these findings, no significant correlation has been found between cow’s milk intake and the onset or recurrence of UC.

On the other hand, β-casomorphin-7 (BCM), one of the main components of cow’s milk protein, has a significant effect on the immune system [15,16]. BCM is a breakdown product of β-casein, primarily derived from type A1 milk in the stomach. Approximately 0.4 g of BCM is produced from 1 L of cow’s milk [17]. BCM consists of seven amino acids (Tyr-Pro-Phe-Pro-Gly-Pro-Ile). Although different isoforms of BCM exist, BCM-7 is primarily released from β-casein and exhibits opioid activity [18]. BCM significantly inhibits DNA synthesis in colonic mucosal lymphocytes stimulated with concanavalin A [15]. Conversely, at low concentrations, BCM inhibits lymphocyte proliferation, but at high concentrations (>10^−7^ M), it stimulates lymphocyte proliferation by activating the T cell helper (Th-2) pathway [16]. Approximately 95% of BCM is degraded in the digestive fluids at the intestinal brush border [19], so under physiological conditions, BCM is present at low concentrations and may exert immunosuppressive effects. Conversely, elevated BCM levels indicate immunostimulatory properties. Previous studies have demonstrated that colon carcinogenesis can be suppressed by inhibiting CD10 (also known as neprilysin), an enzyme expressed in the intestinal mucosa that is involved in the degradation of BCM [20]. However, the immunostimulatory effects of BCM may exacerbate immune dysregulation in UC patients.

Furthermore, abnormalities in intestinal flora are associated with the onset and exacerbation of UC [21]. Antimicrobial peptides play an important role in forming the intestinal mucosal barrier and maintaining the intestinal flora [22,23], whose impairment is associated with the exacerbation of UC [24]. Impaired energy production in the colonic mucosal epithelium may cause dysbiosis of the intestinal flora, which may worsen UC [25,26]. However, the effect of BCM on the intestinal mucosal epithelium is not clear.

In this way, cow’s milk has the potential to activate mucosal immunity and worsen the condition of UC due to its component BCM. This study aims to investigate the effects of BCM on intestinal mucosal immunity and epithelial function and to elucidate the effects of BCM on UC.

## 2. Materials and Methods

### 2.1. Animals and Reagents

C57BL/6 mice (male, 5 weeks old) were purchased from SLC Japan (Shizuoka, Japan). The animals were maintained in a pathogen-free animal facility under a temperature of 23 °C, 50% humidity, and a 12 h light/12 h dark cycle. The animal study was conducted in accordance with the institutional guidelines approved by the Committee for Animal Experimentation of Nara Medical University, Kashihara, Japan, following the current regulations and standards of the Japanese Ministry of Health, Labor and Welfare (approval nos. 11365 and 11528, 11 December 2009). Animals were acclimated to their housing for seven days before the start of the experiment. Mice were fed with a CE-2 standard diet (CLEA Japan, Inc., Tokyo, Japan). BCM was obtained from the Peptide Institute (Ibaraki, Japan); TOP from Sigma-Aldrich Chemical Co. (St. Louis, MO, USA); and alvimopan from 1 nM, DOR inhibitor (Selleck Chemicals, Houston, TX, USA).

### 2.2. Mouse Non-Cancer Model

BALB/c mice (male, 5-week-old) were administered BCM (10 or 20 mg/kg body weight, through gavage) and thiorphan (TOP, 1 mg/kg body weight, intraperitoneal injection) once a day. After 5 days of treatment, the mice were euthanized.

### 2.3. Mouse DSS Colitis Model

C57BL/6 mice (male, 5 weeks old, SLC Japan, 5 mice in each group) were administered BCM (1 mg/mL, free drink, for 28 days) and DSS (3%, free drink, for 14 days, MP Biomedicals, Irvine, CA, USA) and/or TOP (0.1 mg/mL, free drink, for 28 days). After euthanasia, the whole colon was removed, flushed with normal saline, and opened from the cecum to the anus. For the analysis of protein production, the colon mucosa were scraped and frozen under liquid nitrogen to store at −80 °C for further analysis.

### 2.4. Protein Extraction

To prepare whole cell lysates, cells (1 × 10^7^) were washed twice with cold PBS and harvested. Colon mucosa were washed with cold PBS and pelleted with a sonicator (QSONICA, WakenBtech Co., Ltd., Kyoto, Japan). Cells or tissues were lysed with 0.1% SDS-containing RIPA buffer (Thermo Fisher, Tokyo, Japan) [27]. Protein assays were per-formed using a Protein Assay Rapid Kit (Wako Pure Chemical Corporation, Osaka, Japan).

### 2.5. Spleen Cell Isolation

The spleen was minced with a scalpel in HBSS (5 mL, WAKO). DNase (20 μg/mL, Sigma) solution containing 1% FBS (WAKO) was added to the HBSS and incubated at 37 °C for 30 min at room temperature. EDTA (1 mM/mL, WAKO) was added to stop the enzymatic reaction. The cell suspension was filtered with a 0.22 μm filter (Merck, Tokyo, Japan). Cells were washed with 10 mL PBS three times. The suspension was centrifuged at 500× *g* for 5 min at 4 °C and we discarded the supernatant. Red blood cells were lysed by RBC lysis buffer (Funakoshi, Tokyo, Japan). Cells were centrifuged at 500× *g* for 5 min at 4 °C and suspended with cold PBS (1 × 10^8^/mL).

### 2.6. In Vitro Activation of T Cells

Spleen cells were passed through a SepMate-50 column (Stemcell Technologies, Cambridge, MA, USA) with SepMate Medium (Stemcell). After preparation, we resuspend the cells at 5 × 10^7^ cells/mL in SepMate Medium. CD8+ T cells were isolated from the suspended cells by an EasySep Mouse CD8+ T Cell Isolation Kit (Veritas, Santa Clara, CA, USA) according to the manufacturer’s protocol. Briefly, non-CD8+ T cells were labeled for removal with a biotinylated antibody and streptavidin-coated magnetic particles (RapidSpheres™). The labeled cells were separated without a column using an EasySep™ magnet. CD8+ T cells were stimulated in vitro with plate-bound anti-CD3 (2 μg/mL; BD Biosciences, Franklin Lakes, NJ, USA) and soluble anti-CD28 (1 μg/mL; BD Biosciences) and expanded in culture medium containing mouse IL2 (10 ng/mL, BioLegend, San Diego, CA, USA) for 4 days with or without BCM (40 μg/mL).

### 2.7. ELISA

ELISA kits were used for measuring protein levels of CD45RO, CD20, CD3, CD4, CD8, Foxp3, IFNγ, Granzyme B, and α-defensin (Table 1). The assay was performed according to the manufacturer’s instructions, and whole cell lysates were used for the measurements.

### 2.8. Mitochondrial Imaging

Mitochondrial functions were examined using fluorescent probes. Cells were incubated with the probes for 30 min at 37 °C and then photographed using an All-in-One fluorescence microscope (KEYENCE). We used OxiORANGE (10 μM, Goryo Chemicals, Sapporo, Japan) to assess mitochondrial hydroxyradicals (mtROS), mito-Green (100 nM, PromoCell GmbH, Heidelberg, Germany) to assess mtVol, tetra-methylrhodamine ethyl ester (TMRE) (200 nM, Sigma-Aldrich) to assess MMP, and a mitophagy Detection Kit (Dojindo, Kumamoto, Japan), according to the manufacturer’s instructions.

### 2.9. Cell Lines

The IEC6 mouse intestinal epithelial cell line was a gift from Professor Isaiah J Fidler (MD Anderson Cancer Center, Houston, TX, USA). Cells were cultured in Dulbecco’s modified Eagle’s medium (Wako) supplemented with 10% fetal bovine serum (Sigma) at 37 °C in 5% CO_2_.

### 2.10. Flow Cytometry

To demonstrate CD8 + IFNγ+ T cells among the colon mucosa of the DSS colitis model and BCM-treated CD8+ cells, cell surface markers were analyzed by flow cytometry (FACSCalibur, Becton Dickinson, Franklin Lakes, NJ, USA). Colon mucosa was scraped by razor blades and washed with cold PBS. Then, mucosal tissues were squeezed through a 70 μm strainer (Corning Inc., Corning, NY, USA) and filtered through a 30 μm pre-separation filter (Miltenyi Biotech, Bergisch Gladbach, Germany). Single-cell suspensions of CD8+ cells in phosphate-buffered saline (PBS) were exposed to antibodies directly coupled with a fluorochrome for 30 min on ice. The antibodies used were anti-mouse IFNγ and mouse CD8 (Becton Dickinson) coupled to phycoerythrin.

### 2.11. Patients

We obtained frozen tissue samples from 10 patients with UC of the rectal left-colonic type, who were diagnosed at the Department of Molecular Pathology, Nara Medical University, from 2015 to 2022. In addition to 5 biopsies from the colorectum for the evaluation of UC, 3 biopsies were taken from the terminal ileum. One ileal biopsy was used for tissue diagnosis, and two were fresh frozen and used for DNA and protein extraction. Similar biopsies were performed on the same UC patient during the active and remission phases. As written informed consent was not obtained from the patients for their participation in the present study, all identifying information was removed from patient samples prior to their analysis to ensure strict privacy protection (unlinkable anonymization). Thus, written informed consent was not obtained. All procedures were performed in accordance with the Ethical Guidelines for Human Genome/Gene Research enacted by the Japanese Government and with the approval of the Ethics Committee of Nara Medical University (approval number 937, 20 October 2014).

### 2.12. Methylation-Specific PCR

Genomic DNA was extracted from biopsied colonic mucosa using NucleoSpin^®^ DNA RapidLyse (TAKARA, Kyoto, Japan). Mucosal tissue (30 mg) was lysed with the lysis buffer (150 μL) and proteinase K (10 μL), and incubated at 56 °C for 1 h with shaking. The lysed solution was added to a binding buffer (440 μL) and loaded onto the column. The column was spun at 11,000× *g* for 1 min and washed washing buffer (500 μL) twice. The column was spun at 11,000× *g* for 1 min and dried. DNA was eluted from the column with elution buffer (100 μL) by spinning at 11,000× *g* for 1 min. A total of 250 ng of genomic DNA was modified using an Express DNA methylation kit (ENZO Life Science, Farmingdale, NY, USA). The bisulfite-converted DNA was subjected to PCR using the methylated or unmethylated primer set, independently (Table 1). Cycling conditions were 5 min at 95 °C, followed by 42 cycles of 30 sec, 95 °C/30 s, 59 °C/30 s, 72 °C, and 5 min at 72 °C. PCR reactions (15 μL) were analyzed on 2% agarose gel stained with ethidium bromide.

### 2.13. Statistical Analysis

Statistical significance was calculated using a two-tailed Fisher’s exact test and ordinary ANOVA using InStat software (version 3.1, GraphPad, Los Angeles, CA, USA). A two-sided *p* value of <0.05 was considered to indicate statistical significance.

## 3. Results

### 3.1. Effect of BCM in the Mouse Colitis Model

**Figure 1:**
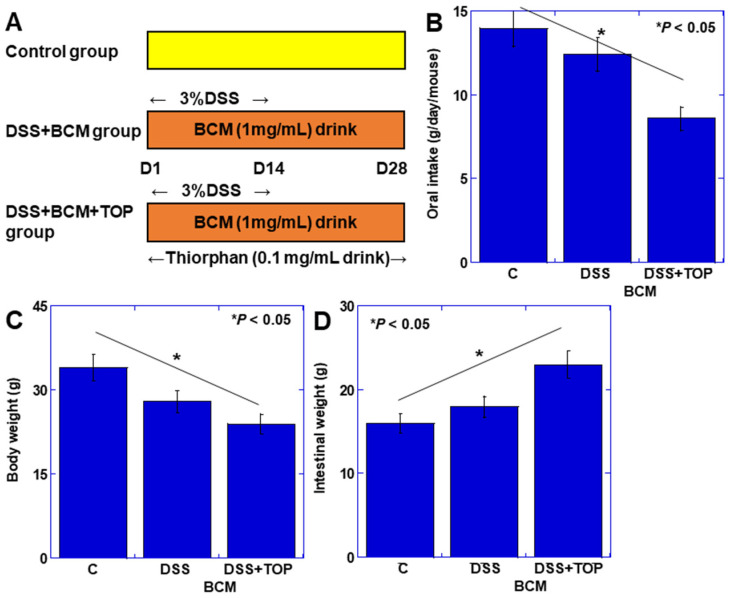
Role of CD10 in BCM-induced mucosal changes in mouse DSS colitis model. (**A**) Experimental protocol. BALB/c mice (*n* = 5) were treated with BCM (20 mg/mL, drinking water), DDS (3% drinking water), and/or TOP (0.1 mg/mL, drinking water). (**B**) Oral intake. (**C**) Body weight. (**D**) Intestinal weight. Error bars indicate standard deviation from 5 mice. Statistical differences were calculated by ANOVA with Bonferroni correction. BCM, β-casomorphin-7; C, control; DSS, dextran sulfate sodium; TOP, thiorphan.

As in the above experiments, it was found that mucosal immunity was affected by BCM by suppressing CD10 in the intestinal mucosa with TOP. Next, to examine the role of CD10 in BCM-induced lymphocyte-induced changes in intestinal inflammation, we employed a dextran sulfate sodium (DSS)-induced colitis mouse model with CD10 suppression by TOP (Figure 1A). Oral food intake and mouse body weight showed significant reductions in the DDS + TOP + BCM group compared to the DDS + BCM group (Figure 1B,C). Conversely, intestinal weight was notably higher in the DDS + TOP + BCM group than in the DDS + BCM group (Figure 1D).

**Figure 2:**
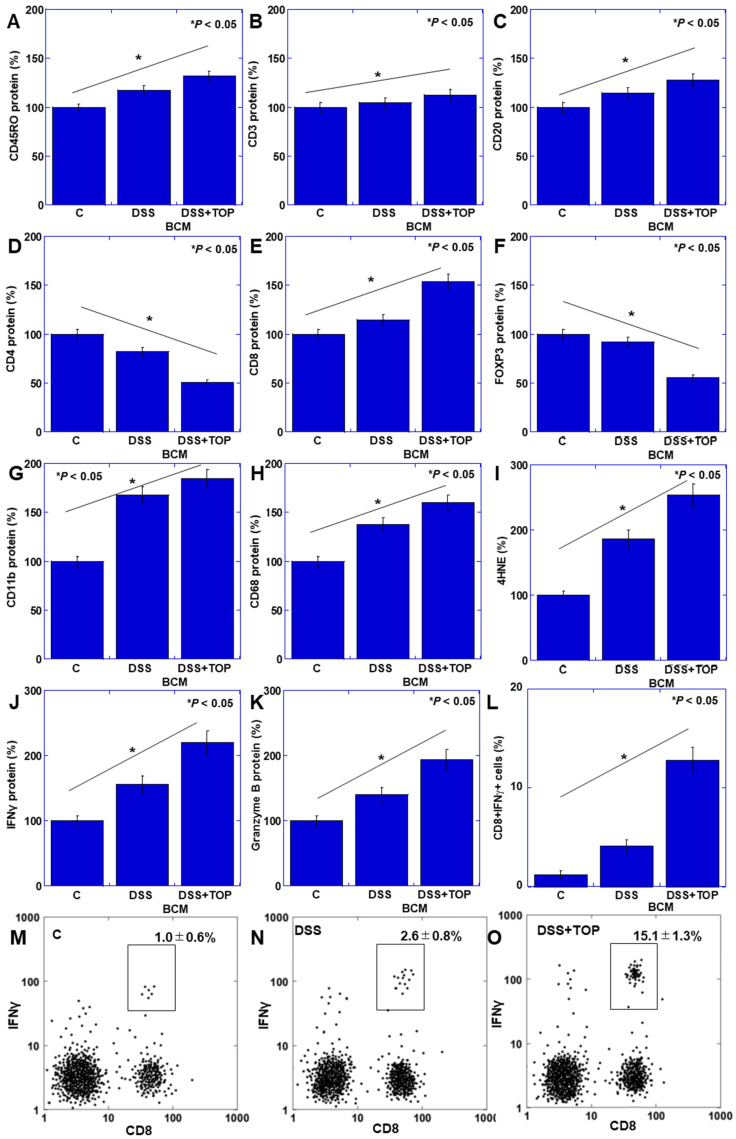
Role of CD10 in BCM-induced lymphocyte changes in mouse DSS colitis model. (**A**–**K**) Lymphocytes markers and other factors were examined in mucosal proteins using ELISA. (**A**) CD11b (neutrophils). (**B**) CD68 (macrophages). (**C**) 4HNE (oxidative stress). (**D**) CD45RO (common lymphocytes). (**E**) CD3. (**F**) CD20. (**G**) CD4. (**H**) CD8. (**I**) FOXP3 (regulatory T). (**J**) IFNγ. (**K**) Granzyme B. (**L**–**O**) Mucosal CD8 + IFNγ+ cells analyzed by flow cytometry. Error bars indicate standard deviation from 5 mice. Statistical differences were calculated by ANOVA with Bonferroni correction. BCM, β-casomorphin-7; C, control; TOP, thiorphan; DSS, dextran sulfate sodium; FOXP3, forkhead box P3; 4HNE, 4-hydroxynonenal; IFNγ, interferon-γ.

The levels of CD11b and CD68, indicative of innate immunity and oxidative stress (4-hydroxynonenal, 4HNE), exhibited significant increases in the DDS + TOP + BCM group compared to the DDS + BCM group (Figure 2A–C). Furthermore, the levels of lymphocytes (CD45RO), CD3, CD20, and CD8 were significantly elevated in the DDS + TOP + BCM group compared to the DDS + BCM group (Figure 2D–F,H). In contrast, the counts of CD4+ and Treg decreased (Figure 2G,I). Additionally, the levels of interferon (IFN)-γ and granzyme B were markedly increased in the DDS + TOP + BCM group relative to the DDS + BCM group, indicating CD8 activation (Figure 2J,K). To confirm activated CD8T cells, CD8 + IFNγ+ cells were measured by flow cytometry (Figure 2L–O). As a result, CD8 + IFNγ+ cells increased 2.6-fold in the DSS+BCM group, but increased 15-fold in the DSS + TOP + BCM group.

### 3.2. Activation of CD8+ T Cells by BCM

**Figure 3:**
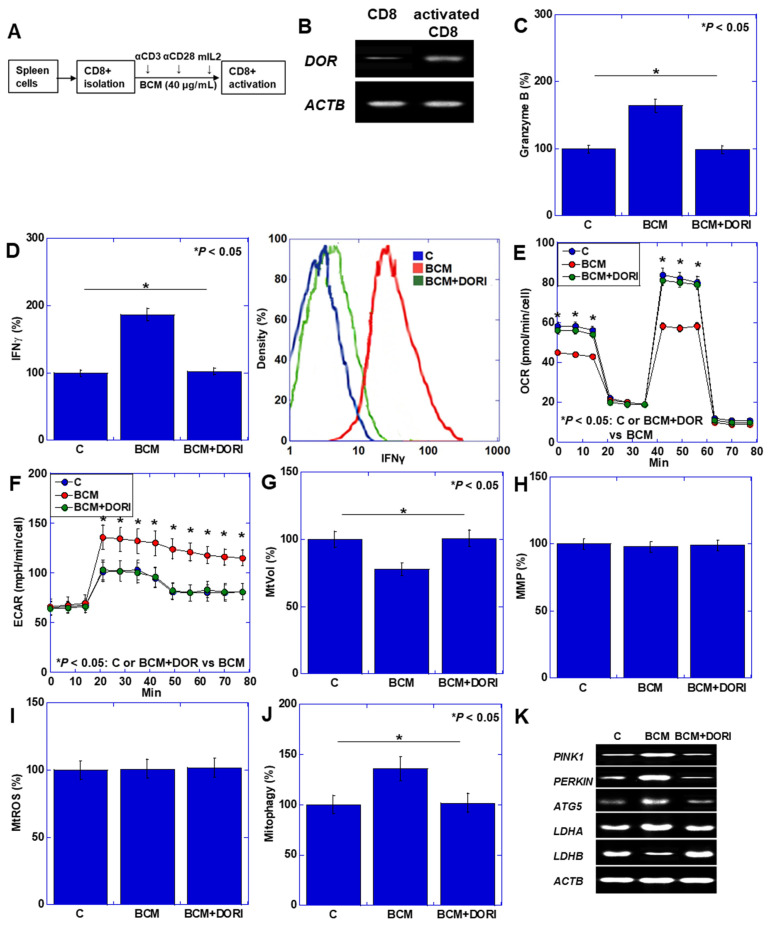
Effect of BCM on activation of CD8+ T cells. (**A**) Protocol of in vitro activation of CD8+ T cells. (**B**) Effect of BCM on expression of DOR in activated CD8+ cells. (**C**,**D**) Granzyme B, IFNγ in cultured medium. Right panel shows flow cytometry of IFNγ+ cells in CD8+ cells. (**E**–**G**) MtVol (**E**), MMP (**F**), and mtROS (**G**). (**H**,**I**) OCR and ECAR. (**J**) Mitophagy. (**K**) Expression of mitophagy- and glycolysis-associated genes. Error bars indicate standard deviation from 3 independent trials. Statistical difference was calculated by ANOVA with Bonferroni correction. αCD3, anti-CD3 antibody; αCD28, anti-CD28 antibody; mIL2, mouse interleukin-2; DOR, δ-opioid receptor; ACTB, actin β; BCM, β-casomorphin-7; DORI, inhibitor (alvimopan); IFNγ, interferon-γ; OCR, oxygen consumption rate; ECAR, extracellular acidification rate; MtVol, mitochondrial volume; MMP, mitochondrial membrane potential; MtROS, mitochondrial reactive oxygen species (mitochondrial hydroxyradical); PINK1, PTEN-induced kinase 1; ATG5, autophagy-related 5; LDH, lactate dehydrogenase.

The above experiments suggested that BCM activates CD8+ T cells under conditions where CD10 is suppressed. Subsequently, we investigated the direct effect of BCM on CD8+ T cell activation in an in vitro model (Figure 3). Activated CD8+ T cells were isolated from mouse spleens, as depicted in Figure 3A. The expression of the δ-opioid receptor (DOR), a receptor for BCM, was initially low in CD8 cells before activation, but it increased upon activation (Figure 3B). The levels of IFNγ and Granzyme B, markers of CD8 activation, were augmented by BCM treatment, while the DOR inhibitor (alvimopan, ALV) nullified the effect of BCM (Figure 3C,D). Notably, BCM treatment led to a reduction in mitochondrial volume (Figure 3E) yet maintained mitochondrial membrane potential (MMP) (Figure 3F), accompanied by an increase in mitochondrial reactive oxygen species (ROS) (Figure 3G). Conversely, BCM suppressed oxidative phosphorylation (OXPHOS) and promoted glycolysis in CD8+ cells (Figure 3H,I). Moreover, increased expression of PTEN-induced kinase 1 (PINK1), PARKIN, and autophagy-related 5 (ATG5), alongside mitophagy, was observed in response to BCM (Figure 3J,K). Additionally, an increase in lactate dehydrogenase A (LDHA) and a decrease in LDHB were noted. In summary, BCM suppresses OXPHOS by promoting mitophagy while concurrently enhancing glycolysis, lactic acid fermentation, and CD8 activation.

### 3.3. Effects of BCM on Intestinal Epithelium

**Figure 4:**
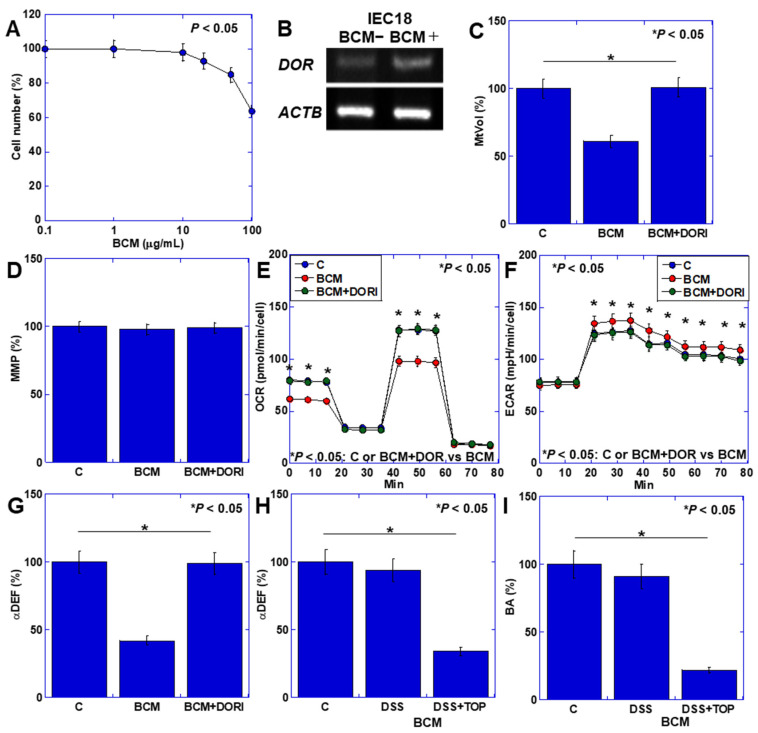
Effect of BCM on intestinal epithelial cells. (**A**) Effect of BCM on cell growth in IEC6 mouse intestinal cells. (**B**) DOR expression in IEC6 cells. (**C**–**G**) Effect of BCM with or without DORI on mtVol (**C**), MMP (**D**), OCR (**E**), ECAR (**F**), and αDEF (**G**). (**H**,**I**) Fecal concentration of αDEF (**H**) and BA (**I**) in DSS colitis model shown in Figure 3 and Figure 4. Error bars indicate standard deviation from 3 independent trials or from 5 mice. Statistical difference was calculated by ANOVA with Bonferroni correction. BCM, β-casomorphin-7; DOR, δ-opioid receptor; DORI, interferon-γ inhibitor (alvimopan); MtVol, mitochondrial volume; MMP, mitochondrial membrane potential; OCR, oxygen consumption rate; ECAR, extracellular acidification rate; αDEF, α-defensin; BA, butyric acid; DSS, dextran sulfate sodium.

Hence, we investigated the effects of BCM on the antimicrobial barrier, recognizing its pivotal role in the development and progression of UC, where the relationship between the mucosa and intestinal microbiota is crucial [28,29]. Subsequently, we delved into the effects of BCM on the intestinal epithelium (Figure 4). Mouse small intestinal epithelial cells IEC6 exhibited the expression of the δ-opioid receptor (DOR), which was enhanced following BCM treatment (Figure 4A). Notably, BCM induced concentration-dependent growth inhibition (Figure 4B). Moreover, BCM led to a reduction in mitochondrial volume while preserving MMP (Figure 4C,D). Furthermore, BCM decreased OXPHOS but enhanced glycolysis (Figure 4E,F). The assessment of α-defensin (αDEF) expression, a vital antibacterial peptide maintaining mucosal–bacterial flora balance, revealed a decrease upon BCM treatment (Figure 4G). In the mouse colitis model depicted in Figure 3, fecal αDEF was significantly lower in the DDS + TOP + BCM group compared to the DDS + BCM group (Figure 4H). Furthermore, the fecal concentration of butyric acid (BA), a product of normal intestinal flora, was also diminished (Figure 4I).

### 3.4. CD10 Status in Human UC Cases

**Figure 5:**
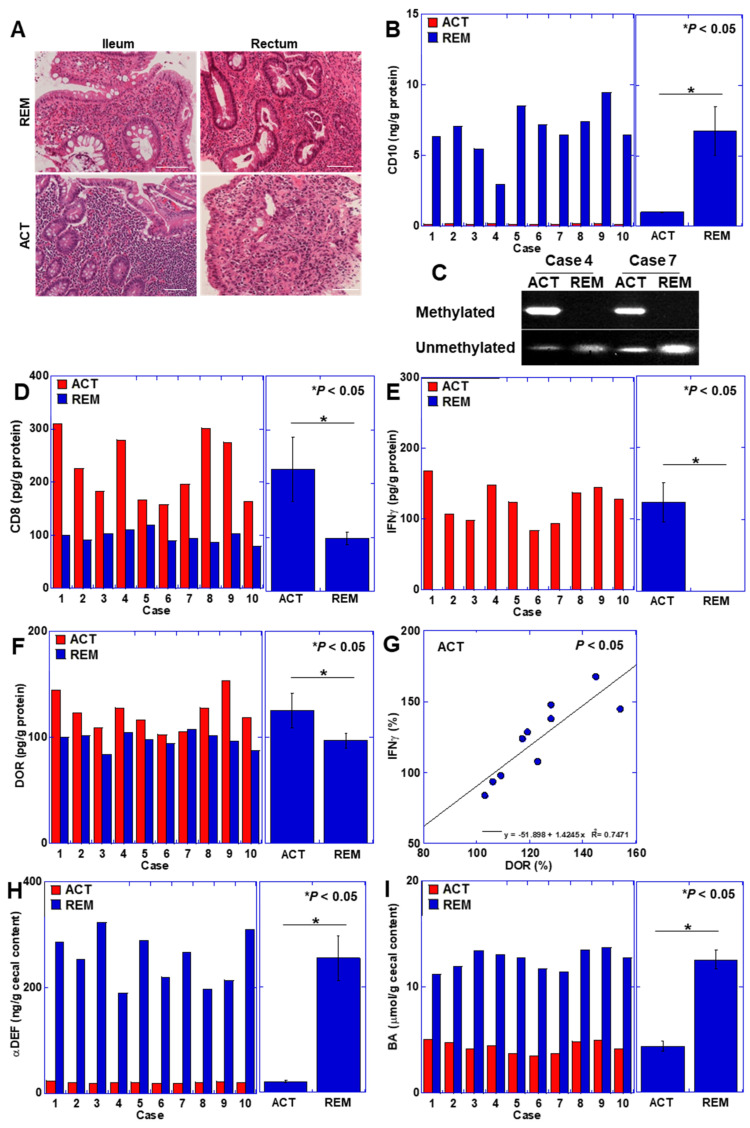
CD10 status in 10 human UC cases. The ileal mucosa of the active and remission phases of UC in the same case were compared. (**A**) Histological appearance of ileal and rectal mucosa in case 1. Scale bar, 50 μm. (**B**) Comparison between the ileal mucosa of the active and remission phases of UC on CD10 protein. (**C**) MSP on CD10 promoter. (**D**–**F**) Comparison between the ileal mucosa of the active and remission phases of UC on CD8 (**D**), IFNγ (**E**), and DOR (**F**). (**G**) Comparison of protein levels between DOR and IFNγ. (**H**,**I**) Comparison between the ileal mucosa of the active and remission phases of UC on fecal αDEF (**H**) and BA (**I**). Error bars indicate standard deviation from 3 independent trials or from 10 cases. Statistical differences were calculated by ANOVA with Bonferroni correction. UC, ulcerative colitis; ACT, active phase; REM, remission phase; methylated, methylated CpG-specific primer; unmethylated, unmethylated CpG-specific primer; IFNγ, interferon-γ; DOR, δ-opioid receptor; αDEF, α-defensin; BA, butyric acid.

Lastly, we explored the effect of CD10 on human UC patients. It was difficult to clarify BCM intake directly in patients. Therefore, we examined whether the changes seen so far in this study, such as CD10 suppression and CD8+ lymphocyte activation, were observed in UC patients. In order to examine changes in the small intestinal mucosa, where CD10, which degrades BCM, is expressed at high levels on the mucosal brush border, we examined the ileal mucosa, which is anatomically distant from the active lesions of UC. Ileal mucosal changes were compared between active and remission stages in 10 cases of left colorectal-type or rectal-type UC within the same patient (Figure 5). During the active phase, a lesion with a Geboes histopathology score of 5 was evident in the rectum; however, during remission, the lesion regressed to approximately a Geboes histopathology score of 2 (case 1) (Figure 5A). Interestingly, the ileal mucosa, which is distant from the active lesions of UC, also showed a stronger inflammatory cell infiltrate in the active phase of UC than in the remission phase. Additionally, in the active phase of the ileal mucosa, a decrease in CD10 protein levels was observed (Figure 5B). The assessment of CpG methylation of the CD10 gene promoter revealed methylation in both cases (Figure 5C). Intramucosal CD8 cells and IFNγ levels were elevated in active-phase mucosa (Figure 5D,E). Moreover, the level of DOR was augmented in active-phase mucosa and correlated with IFNγ levels (Figure 5F,G). Furthermore, intramucosal αDEF and BA concentrations were diminished in active-phase mucosa (Figure 5H,I). These data are comparable to the alterations in intestinal immunity induced by oral BCM administration under CD10 suppression in the intestinal mucosa in the mouse colitis model.

## 4. Discussion

In this study, we investigated the impact of CD10 on the effect of BCM on mucosal immunity. Our findings revealed that BCM results in reprogramming of the energy metabolism of CD8 lymphocytes from OXPHOS to glycolysis and lactic acid fermentation, leading to the activation of CD8 cells under CD10-suppressed conditions. Additionally, in the intestinal epithelium, BCM was found to suppress energy metabolism and antimicrobial peptide production, thereby altering the intestinal flora. Similar observations were made in the mucosal tissues of patients with active UC, where the underlying mechanism involved the epigenetic suppression of CD10 expression in the intestinal epithelium.

Our data indicated that BCM led to a reduction in mitochondrial OXPHOS while promoting mitophagy; however, no decrease in MMP or increase in mitochondrial ROS was observed. Notably, the activation of DOR has been reported to have protective effects on myocardial and nerve cells while concurrently promoting mitophagy and enhancing mitochondrial quality control [30,31,32]. This dual role of DOR activation has been associated with the suppression of apoptosis [33,34].

We demonstrated that BCM induces a shift in cellular energy metabolism from OXPHOS to glycolysis–lactic acid fermentation, consequently activating CD8+ lymphocytes. T lymphocytes typically rely on OXPHOS through fatty acid oxidation in naïve T cells, while effector T cells favor glycolysis–lactic acid fermentation [35]. The inhibition of OXPHOS has been shown to activate effector T cells [36,37], consistent with our findings. This metabolic modulation by BCM is believed to contribute to its promotion of antitumor immunity [20].

Furthermore, our study revealed that BCM reduces antimicrobial peptide production by altering the energy metabolism of intestinal epithelial cells. Antimicrobial peptides play crucial roles in microbiota regulation and the maintenance of intestinal flora balance [25]. Disruptions in the intestinal flora and compromised mucosal barrier function are significant factors in the initiation and exacerbation of UC [29]. Dysbiosis resulting from impaired mitochondrial energy metabolism in colonic epithelial cells exacerbates UC [26]. We then measured BA, a marker of normal gut flora and an intact mucosal barrier [38]. Decreased antimicrobial peptides and BA in the UC cases suggested abnormalities in the intestinal flora and a compromised mucosal barrier, which correlated with active inflammation in UC.

Our findings from a mouse colitis model suppressing CD10 indicate that BCM may induce similar alterations in the intestinal flora, exacerbating UC. BCM triggers the reprogramming of energy metabolism in intestinal epithelial cells and CD8+ cells through the activation of DOR. This metabolic shift likely leads to a negative energy balance in the intestinal epithelium and reduces the production of antimicrobial peptides, further contributing to dysbiosis and the exacerbation of UC.

It is crucial to note that under normal physiological conditions, orally administered BCM undergoes degradation by CD10, which is abundantly expressed in the intestine and typically does not induce significant alterations in lymphocytes or the intestinal epithelium [20,39]. In our experimental setup, we utilized TOP to suppress CD10 activity, thereby inducing BCM-associated changes. Additionally, it is noteworthy that CD10 expression is epigenetically suppressed in the ileal mucosa of patients experiencing active-phase UC. Chronic inflammation can trigger CpG island methylation [27,40], leading to the downregulation of CD10 expression. While UC primarily affects the colon, nonspecific inflammation can extend to the small intestinal mucosa [41], resulting in reduced CD10 expression. Previous studies have indicated a correlation between UC activity and decreased intramucosal CD10 levels [42]. Our findings suggest that diminished CD10 expression in the ileal mucosa correlates with UC activity. Active UC lesions may provoke nonspecific inflammation in the intestinal mucosa, potentially instigating epigenetic alterations.

CD10 functions as an endopeptidase, targeting substrates including peptides implicated in intestinal inflammation, such as BCM, substance P, vasoactive intestinal peptide, and cholecystokinin [20,43,44,45]. Reduced CD10 activity may result in the accumulation of these pro-inflammatory substances within the intestinal tract, potentially worsening UC [46]. Furthermore, CD10 plays a role in degrading enkephalins, which possess anti-inflammatory properties [39,47]. However, patients with UC often exhibit decreased blood enkephalin levels, possibly due to heightened enkephalinase activity [48]. The suppression of CD10 expression in UC is considered an exacerbating factor, with decreased CD10 expression in mucosal tissues potentially serving as an indicator of disease activity or relapse. The weakest aspect of this study is that we were unable to directly assess BCM intake in UC patients. Future prospective studies should examine direct associations between BCM intake and UC activity. Therefore, comprehensive clinical investigations are also necessary to further elucidate the relationship between mucosal CD10 expression and UC activity.

Until now, the relationship between cow’s milk and UC has been unclear, but the relationship between BCM and CD10 revealed that milk is not associated with the onset of UC, but may exacerbate the disease during the active stage. This finding may help avoid excessive avoidance of cow’s milk in UC patients and lead to appropriate dietary management.

In this study, only male mice were used. However, recent studies have pointed out that females have fewer physiological fluctuations than males and are better at extrapolating to humans [49]. Gender differences have also been observed in the response to the stimulation of opioid receptors [28,50]. Considering this point, in the future, when examining the effects of BCM, it will be necessary to use an experimental system using both male and female rodents, or to examine gender differences in UC patients

Regarding the limitations of our study, it should be raised that we could only partially confirm mucosal lymphocyte surface markers by flow cytometry. Further examination using flow cytometry will enable us to clarify in more detail our findings on BCM-associated lymphocyte alteration. A decrease in αDEF was observed as one of the changes in the mucosal epithelium of BCM. In addition, a decrease in BA, which is one of the indicators of the state of the intestinal flora, was observed. These suggest changes in the intestinal flora due to BCM. However, we were unable to examine the actual bacterial species of the intestinal flora. This point suggests that our study remains in the realm of speculation. In the future, it will be necessary to identify changes in bacterial species in the intestinal flora to further elucidate the relationship between BCM-CD10 and UC. 

In this study, we reported the importance of intestinal CD10 in mucosal immunity, which is influenced by BCM, a milk component. CD10 expression is epigenetically suppressed by persistent inflammation. In this respect, CD10 is a molecule that is prone to abnormal expression in UC, leading to the abnormal activation of peptides such as BCM, which are degraded by CD10, and resulting in the exacerbation of UC activity. The action of BCM leads to exacerbation in UC, but suppression in cancer. Targeting the enzymatic activity and expression of CD10 is important in the treatment of these diseases by modulating the role of BCM.

## Figures and Tables

**Table 1:** PCR primers and ELISA kits.

**RT-PCR Primers**				
**Gene**	**Species**	**ID**	**Upper**	**Lower**
PINK1	mouse	NM_026880.2	ccatcgggatctcaagtccg	agagccaggcgatcatcttg
Parkin	mouse	AB019558.1	cctgcaaacaagcaaccctc	gtcccggcagaaaacaaacc
ATG5	mouse	MG656078.1	tgtgcttcgagatgtgtggt	ttctggatgaaaggccgctc
LDHA	mouse	BC094019.1	gcatggcagcctcttcctta	ccaagtctgccacagagagg
LDHB	mouse	BC046755.1	gactccgaaaattgtggccg	tccgggagaggtttttcagc
**MSP Primers**				
**Gene**	**Species**	**ID**	**Upper**	**Lower**
CD10 (methylated)	human	NG_051105.1	agaaaacggagcatccgaca	ctgcaagagcagagaaaaca
CD10 (unmethylated)	human	NG_051105.1	agaaaatggagcatctgaca	ctacaagaacagagaaaaca
**ELISA**				
**Target**	**Species**	**Cat#**	**Company**	
CD45RO	mouse	EM14RB	Thermo Fisher, Tokyo, Japan	
CD45RO	human	65150-1	Proteintech, Rosemont, IL, USA	
CD20	mouse	LS-F49371	LSbio, Shirley, MA, USA	
CD20	human	ab285273	Abcam, Waltham, MA, USA	
CD3	mouse	NBP2-75137	Novus Biologicals, LLC, Centennial, CO, USA	
CD3	human	CRS015-C02	Acro Biosystems, Tokyo, Japan	
CD4	mouse	NBP2-75148	Novus Biologicals, LLC, Centennial, CO, USA	
CD4	human	EH90RB	Thermo Fisher, Tokyo, Japan	
CD8	mouse	NBP2-78729	Novus Biologicals, LLC, Centennial, CO, USA	
CD8	human	LS-F26719	Thermo Fisher, Tokyo, Japan	
FoxP3	mouse	ab289645	Abcam, Waltham, MA, USA	
FoxP3	human	22228-1-AP	Proteintech, Rosemont, IL, USA	
IFNγ	mouse	KMC4021	Thermo Fisher, Tokyo, Japan	
IFNγ	human	EHIFNG	Thermo Fisher, Tokyo, Japan	
Granzyme B	mouse	BMS6029	Thermo Fisher, Tokyo, Japan	
Granzyme B	human	ab46142	Abcam, Waltham, MA, USA	
αDefensin	mouse	EK11275	Signalway Antibody, Greenbelt, MA, USA	
αDefensin	human	EL006657HU	Cusabio, Houston, TX, USA	
4HNE	-	ab238538	Abcam, Waltham, MA, USA	
Butyric acid	-	abx258338	Abbexa Ltd., Cambridge, UK	

PINK1, PTEN-induced kinase 1; ATG5, autophagy-related 5; LDH, lactate dehydrogenase; FOXP3, forkhead box P3; IFNγ, interferon-γ; 4HNE, 4-hydroxynonenal.

## Data Availability

The data are contained within the article.

## Abbreviations

BCM	β-casomorphin-7
TOP	thiorphan
OXPHOS	oxidative phosphorylation
UC	ulcerative colitis
Treg	regulatory T cells
DSS	dextran sulfate sodium
4HNE	4-hydroxynonenal
IFN	interferon
ALV	alvimopan
ROS	reactive oxygen species
PINK1	PTEN-induced kinase 1
ATG5	autophagy-related 5
LDH	lactate dehydrogenase
DOR	δ-opioid receptor
αDEF	α-defensin
BA	butyric acid
